# Development and validation of a nomogram for predicting rapid relapse in triple-negative breast cancer patients treated with neoadjuvant chemotherapy

**DOI:** 10.3389/fcell.2024.1417366

**Published:** 2024-09-02

**Authors:** Tao Ma, Xin-Yu Liu, Shuang-Long Cai, Jin Zhang

**Affiliations:** ^1^ The Third Department of Breast Cancer, Tianjin Medical University Cancer Institute and Hospital, National Clinical Research Center for Cancer, Tianjin, China; ^2^ Tianjin’s Clinical Research Center for Cancer, Tianjin Medical University Cancer Institute and Hospital, Tianjin, China; ^3^ Key Laboratory of Breast Cancer Prevention and Therapy, Tianjin Medical University, Ministry of Education, Tianjin, China; ^4^ Key Laboratory of Cancer Prevention and Therapy, Tianjin Medical University Cancer Institute and Hospital, Tianjin, China; ^5^ Key Laboratory of Cancer Immunology and Biotherapy, Tianjin, China; ^6^ Department of Breast Surgery, Shengli Clinical Medical College of Fujian Medical University, Fujian Provincial Hospital, Fuzhou University Affiliated Provincial Hospital, Fuzhou, Fujian, China

**Keywords:** triple-negative breast cancer, neoadjuvant chemotherapy, rapid relapse, nomogram, predictive markers

## Abstract

**Background:**

Triple-negative breast cancer (TNBC) accounts for disproportionately poor outcomes in breast cancer, driven by a subset of rapid-relapse TNBC (rrTNBC) with marked chemoresistance, rapid metastatic spread, and poor survival. This study aimed to develop and validate a nomogram based on clinicopathological characteristics to predict rapid relapse in TNBC patients treated with neoadjuvant chemotherapy (NAC) first.

**Methods:**

The clinicopathological data of 504 TNBC patients treated with NAC first in Tianjin Medical University Cancer Hospital were analyzed retrospectively, with 109 rapid relapsed patients, and 395 non-rapid relapsed patients, respectively. Based on clinicopathologic characteristics, and follow-up data were analyzed. The independent predictors of clinicopathological characteristics were identified by logistic regression analysis and then used to build a nomogram. The concordance index (C-index), the area under the curve (AUC) of receiver operating characteristic (ROC), and calibration plots were used to evaluate the performance of the model.

**Results:**

Univariate and multivariate logistic regression analyses showed that age at diagnosis (age≥50 years, OR = 0.325,95% CI:0.137–0.771), Nodal staging (N3 staging, OR = 13.669,95% CI:3.693–50.592),sTIL expression levels (sTIL intermediate expression, OR = 0.272,95% CI:0.109–0.678; sTIL high expression, OR = 0.169,95% CI:0.048–0.594), and NAC response (ORR, OR = 0.059,95% CI:0.024–0.143) were independent predictors of rapid relapse in TNBC patients treated with NAC firstly. Among these independent predictors, age ≥ 50 years, sTIL intermediate expression, sTIL high expression, and ORR in NAC were independent protective factors for rapid relapse in TNBC NAC patients. N3 staging was an independent risk factor for rapid relapse in TNBC NAC patients. The ROC curve, calibration curve, and decision curve analysis were used to validate the model. The C-Index of the training sets and validation sets were 0.938 and 0.910, respectively. The Brier scores of the training sets and validation sets were 0.076 and 0.097, respectively.

**Conclusion:**

This study developed and verified a nomogram for predicting rapid relapse in TNBC NAC patients, and the predictive model had high discrimination and accuracy.

## 1 Introduction

Triple-negative breast cancer (TNBC), accounting for only 10%–15% of molecular subtypes in breast cancer, contributes to over 30%–40% of breast cancer-related deaths (Perou et al., 2000; Harbeck and Gnant, 2017). TNBC has been the most lethal and challenging subtype due to its high tumor heterogeneity, invasiveness, and lack of targetable receptors (estrogen receptor, progesterone receptor, and human epidermal growth factor receptor two are all negative) (Cossetti et al., 2015; Ge et al., 2021; Pan et al., 2017). Compared to other molecular subtypes of breast cancer, TNBC often presents with lower differentiation, higher histologic grade, proliferation rate, and a higher proportion of lymph node metastasis. Distant organ metastases, particularly to the lungs and brain, are more common than bone metastases, showing organ selectivity (Lin et al., 2008; Lin et al., 2012; Haffty et al., 2006; Dent et al., 2009). These factors contribute to poorer disease-free survival and overall survival outcomes in TNBC patients (Agarwal et al., 2016).

As of now, research on the population of TNBC has primarily focused on the overall group at risk of recurrence and metastasis, with little to no further subdivision of this subgroup for recurrent metastatic TNBC. However, in our clinical practice, we often encounter a specific subset of TNBC patients who, once diagnosed, regardless of surgery, even within their standardized diagnostic and treatment processes, exhibit clear signs of chemotherapy resistance, rapid distant organ metastasis, or death within 6 months to 1–2 years post-adjuvant therapy. Currently, there is scarce knowledge about this subgroup, with few or no scholars summarizing or conducting in-depth exploratory studies on its nature.

Combining findings from several large-scale international studies on TNBC cohorts, reports indicate that the median time interval between diagnosis of breast cancer and occurrence of distant metastasis ranges from 19.7 months to 31.2 months, approximately 2 years (Lin et al., 2008; Dent et al., 2007; van Roozendaal et al., 2016; Ghosh et al., 2018). Consequently, to further understand and recognize this subset within TNBC, in our research, we define it as rapid-relapse triple-negative breast cancer (RR-TNBC), characterized by the development of distant organ metastasis or death within 24 months post-diagnosis of breast cancer. This study aims to analyze potential predictive markers for RR-TNBC patients treated with neoadjuvant chemotherapy, using a large sample size.

## 2 Materials and methods

### 2.1 Patients

**FIGURE 1:**
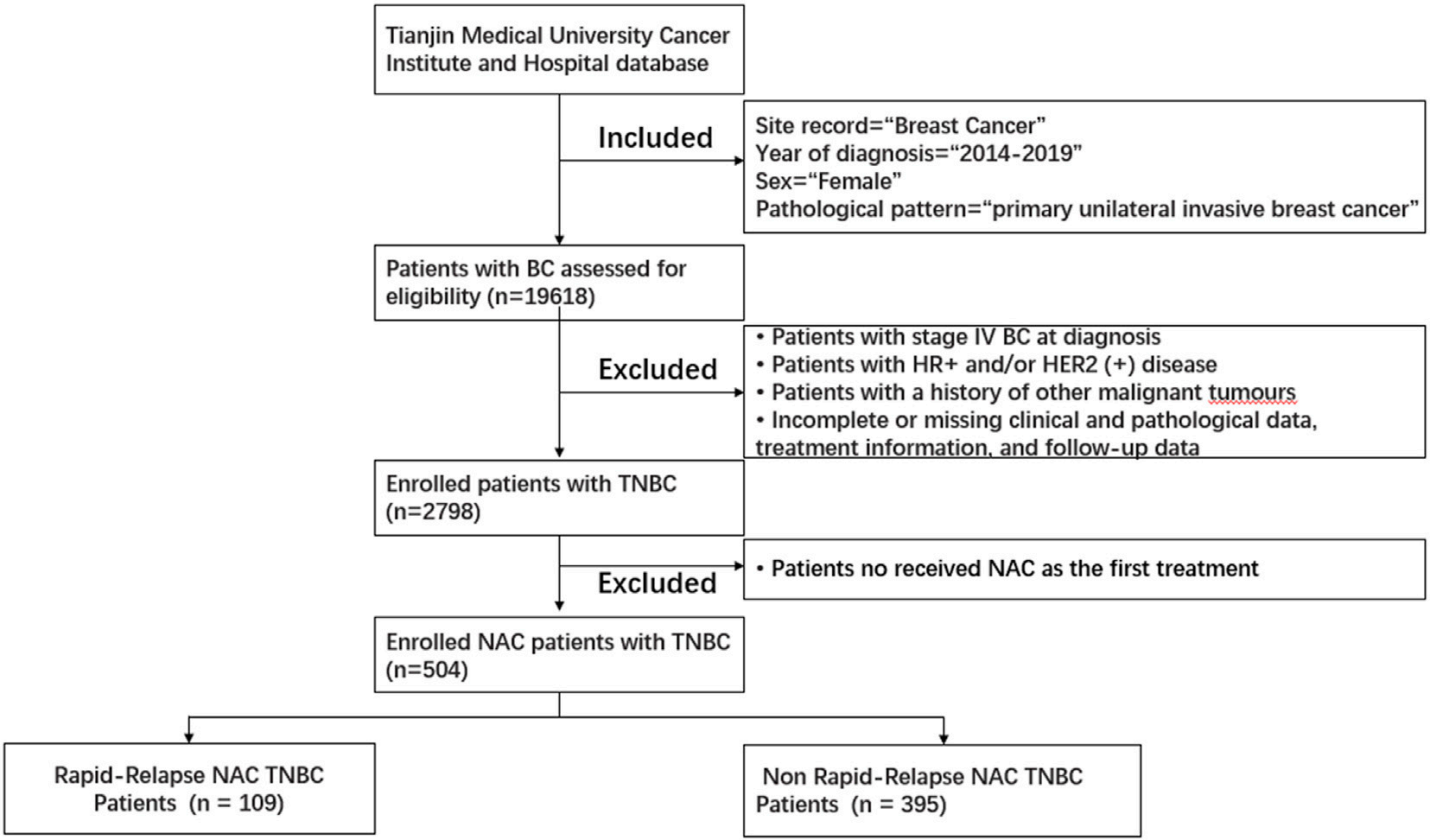
Patient selection flowchart for this study. Abbreviations: HER2 (+), HER2-positive; HR (+), hormone receptor-positive; TNBC, triple-negative breast cancer.

From 1 January 2014, to 31 December 2019, a total of 504 female NAC patients diagnosed with TNBC and treated at Tianjin Medical University Cancer Institute and Hospital were included in the study (Figure 1). Clinical data include age, menstrual status, family history, surgical approach, chemotherapy drugs used during neoadjuvant and adjuvant therapy stages, receipt of postoperative radiotherapy, NAC response, and recurrence/metastasis status. Pathological data include pathological pattern, postoperative pathological T staging, postoperative pathological N staging, postoperative pathological TNM staging, tumor grade, lymph-vascular invasion, stromal tumor-infiltrating lymphocytes (sTIL) expression levels, ER, PR, different HER2 expression levels, Ki-67, P53, CK5/6, EGFR statuses.

Inclusion criteria: (Perou et al., 2000): Diagnosed with primary unilateral invasive breast cancer and received NAC as the first treatment. (Harbeck and Gnant, 2017). Negative status for ER, PR, and Her2. (Cossetti et al., 2015). Underwent surgery followed NAC. Exclusion criteria: (Perou et al., 2000): The initial diagnosis was stage IV breast cancer. (Harbeck and Gnant, 2017).Incomplete or missing clinical and pathological data, treatment information, and follow-up data.

The recurrence/metastasis status includes the following: the time from breast cancer diagnosis to the first occurrence of distant metastasis or death, types of recurrent metastasis (including rapid relapse TNBC and non-rapid relapse TNBC, specifically categorized as follows: using 2 years as the threshold, ≤2 years for the RR-TNBC group where distant metastasis or death occurs; >2 years for the Non-RR-TNBC group including patients with distant metastasis occurring after >2 years and those who have not experienced recurrent metastasis until the follow-up date), and the number of patients for each type of recurrent metastasis.

Immunohistochemistry (IHC) is used to test HER2 status and/or fluorescence *in situ* hybridization (FISH), classified based on the American Society of Clinical Oncology and the College of American Pathologists clinical practice guidelines for HER2 testing of 2013, respectively, and the Belgian Guidelines for HER2 testing (Wolff et al., 2013). HER-2 expression levels in TNBC include IHC 0, IHC 1+, or IHC 2+ with FISH negative. Hormone receptor and Ki67 levels are evaluated using the Allred scoring system (Harvey et al., 1999). P53 expression ≤10% is considered P53 negative, while P53 expression >10% is categorized as P53 positive. The expression levels of cytokeratin 5/6 (CK5/6) and epidermal growth factor receptor (EGFR) < 1% are CK5/6 and EGFR negative, and expression levels ≥1% are considered positive. sTILs are assessed according to internationally recognized criteria. sTILs evaluation is classified into three categories: low (≤10%), intermediate (10% to ≤40%), and high (>40%). All IHC readings are validated by two independent, blinded, and trained pathologists. The NAC response is classified into two groups: the Objective Response Group (ORR) and the No Response Group (NR). The Objective Response Group includes the Complete Response (CR) group and the Partial Response (PR) group. The No Response Group includes the Stable Disease (SD) group and the Progressive Disease (PD) group. The assessment criteria for NAC are mainly based on the Response Evaluation Criteria In Solid Tumors (RECIST), version 1.1.

### 2.2 Follow-up strategy

The follow-up start date is the time of the first BC diagnosis, with a follow-up end date of 1 June 2023. The main follow-up methods include outpatient visits and phone calls. Follow-up content involves monitoring whether there are recurrences in local or regional lymph nodes and distant metastases during regular check-ups.

Local or regional lymph node recurrence refers to the recurrence of the affected side breast or chest wall and regional lymph nodes, while distant metastasis indicates the presence of lesions in distant organs detected through clinical and imaging examinations. Common organs for distant metastasis in breast cancer patients include the lungs, liver, bones, and brain.

Follow-up strategies in our study are as follows: routine breast/liver color ultrasound, ECT, chest and cranium CT plain scan for every initial BC patient to exclude the possibility of distant metastasis. In the first 3 years after BC diagnosis, we regularly reviewed breast/liver color ultrasound, X-ray, or chest CT plain scan every 3 months. Patients who survived 3–5 years after BC diagnosis were regularly reviewed with these items every 6 months. Patients who survived more than 5 years after BC diagnosis were regularly reviewed with these items every 1 year.

### 2.3 Statistical methods

This study utilizes the R statistical software version 4.1.0 (R Project for Statistical Computing) for statistical analysis. One-time random partitioning: the random seed is set at 123456, and the data is randomly split into training and validation sets in a 7:3 ratio. Count data is described using N (%), and intergroup comparisons are conducted using the chi-square test and Fisher’s exact test.

Multifactor logistic regression modeling is performed on the training dataset, incorporating variables with *P* < 0.10 from univariate analysis. Stepwise bidirectional selection is employed to determine the optimal logistic regression model associated with rapid relapse in NAC TNBC based on the training set, using AIC minimization. Visualization is done using a nomogram.

Based on the training data, an optimal logistic regression predictive model related to rapid relapse in NAC TNBC is established. This model is used for prediction on both the training and validation sets, and ROC curves, decision curve analysis (DCA), and calibration plots are generated to evaluate the discrimination and accuracy of the multifactor logistic model using ROC curve and calibration plot assessments.

## 3 Results

### 3.1 Comparison analysis of clinical and pathological parameters in NAC TNBC patients between training and validation sets

Univariate comparison analysis of clinical and pathological parameters in TNBC patients from the training and validation sets revealed no statistically significant differences between the two groups for the following variables: age at breast cancer diagnosis (*P* = 0.427), menstrual status (*P* = 0.323), family history (*P* = 0.815), surgical approach (*P* = 0.704), pathological pattern (*P* = 0.635), postoperative pathological TNM staging (*P* = 0.804), postoperative pathological T staging (*P* = 0.242), postoperative pathological N staging (*P* = 0.152), tumor grade (*P* = 0.216), lymph-vascular invasion (*P* = 0.157), sTIL expression levels (*P* = 0.982), Her2 expression levels (*P* = 0.110), Ki67 expression status (*P* = 0.104), P53 expression status (*P* = 0.324), CK5/6 expression status (*P* = 0.085), EGFR expression status (*P* = 0.512), radiation therapy status (*P* = 0.434), chemotherapy drugs used (*P* = 0.694), and NAC response (*P* = 0.892). The clinical and pathological parameters demonstrated comparability between the training and validation sets (Table 1).

**TABLE 1:** Comparison analysis of clinical and pathological parameters in NAC TNBC patients between training and validation sets.

Variables	Total (n = 504)	Training Sets (n = 352)	Validation sets (n = 152)	*p*-Value
Age at diagnosis				0.427
<50 years	255 (50.60)	174 (49.43)	81 (53.29)	
≥50 years	249 (49.40)	178 (50.57)	71 (46.71)	
Menstrual status				0.323
Premenopausal	285 (56.55)	194 (55.11)	91 (59.87)	
Postmenopausal	219 (43.45)	158 (44.89)	61 (40.13)	
Family history				0.815
No	462 (91.67)	322 (91.48)	140 (92.11)	
Yes	42 (8.33)	30 (8.52)	12 (7.89)	
Surgical approach				0.704
Radical surgery	458 (90.87)	321 (91.19)	137 (90.13)	
Breast-conserving surgery	46 (9.13)	31 (8.81)	15 (9.87)	
Pathological pattern				0.635
Invasive ductal carcinoma	484 (96.03)	339 (96.31)	145 (95.39)	
Metaplastic	11 (2.18)	8 (2.27)	3 (1.97)	
Others	9 (1.79)	5 (1.42)	4 (2.63)	
Postoperative pathological TNM staging				0.804
0 + I	197 (39.09)	135 (38.35)	62 (40.79)	
II	166 (32.94)	119 (33.81)	47 (30.92)	
III	141 (27.98)	98 (27.84)	43 (28.29)	
Postoperative pathological T staging				0.242
T0/Tis + T1	304 (60.32)	215 (61.08)	89 (58.55)	
T2	127 (25.20)	82 (23.30)	45 (29.61)	
T3+T4	73 (14.48)	55 (15.62)	18 (11.84)	
Postoperative pathological N staging				0.152
N0	271 (53.77)	185 (52.56)	86 (56.58)	
N1	112 (22.22)	86 (24.43)	26 (17.11)	
N2	59 (11.71)	36 (10.23)	23 (15.13)	
N3	62 (12.30)	45 (12.78)	17 (11.18)	
Tumor grade				0.216
G1+G2	208 (41.27)	139 (39.49)	69 (45.39)	
G3	296 (58.73)	213 (60.51)	83 (54.61)	
Lymph-vascular invasion				0.157
No	97 (19.25)	62 (17.61)	35 (23.03)	
Yes	407 (80.75)	290 (82.39)	117 (76.97)	
sTIL expression levels				0.982
Low	120 (23.81)	83 (23.58)	37 (24.34)	
Intermediate	143 (28.37)	100 (28.41)	43 (28.29)	
High	241 (47.82)	169 (48.01)	72 (47.37)	
Her2 expression levels				0.110
IHC 0	99 (19.64)	62 (17.61)	37 (24.34)	
IHC 1+	268 (53.17)	197 (55.97)	71 (46.71)	
IHC 2+/Fish-	137 (27.18)	93 (26.42)	44 (28.95)	
Ki67				0.104
Ki67 ≤ 20	119 (23.61)	76 (21.59)	43 (28.29)	
Ki67>20	385 (76.39)	276 (78.41)	109 (71.71)	
P53				0.324
Negative	275 (54.56)	187 (53.12)	88 (57.89)	
Positive	229 (45.44)	165 (46.88)	64 (42.11)	
CK5/6				0.085
Negative	127 (25.20)	81 (23.01)	46 (30.26)	
Positive	377 (74.80)	271 (76.99)	106 (69.74)	
EGFR				0.512
Negative	123 (24.40)	83 (23.58)	40 (26.32)	
Positive	381 (75.60)	269 (76.42)	112 (73.68)	
Radiation therapy status				0.434
No	173 (34.33)	117 (33.24)	56 (36.84)	
Yes	331 (65.67)	235 (66.76)	96 (63.16)	
Chemotherapy drugs used				0.694
Anthracyclines	3 (0.60)	2 (0.57)	1 (0.66)	
Taxanes	22 (4.37)	16 (4.55)	6 (3.95)	
Anthracyclines + taxanes	357 (70.83)	244 (69.32)	113 (74.34)	
Combined with platinum	122 (24.21)	90 (25.57)	32 (21.05)	
NAC response				0.892
NR	158 (31.35)	111 (31.53)	47 (30.92)	
ORR	346 (68.65)	241 (68.47)	105 (69.08)	

Family history, HBOC-related cancer history.

CK5/6: cytokeratin 5/6; HER2: human epidermal growth factor receptor 2.

FISH, fluorescence in situ hybridization; EGFR, epidermal growth factor receptor Objective ORR, Response Group; NR, No Response Group.

### 3.2 Univariate analysis of clinical and pathological parameters between rapid relapse and non-rapid relapse NAC TNBC patients in the training set

The univariate analysis revealed statistically significant differences between rapid relapse and non-rapid relapse NAC TNBC patients in the training set for the following variables: postoperative pathological TNM staging (*P* < 0.001), postoperative pathological T staging (*P* < 0.001), postoperative pathological N staging (*p* < 0.001), sTIL expression (*P* < 0.001), and NAC response (*P* < 0.001) (Table 2).

**TABLE 2:** Univariate analysis of clinical and pathological parameters between rapid relapse and non-rapid relapse NAC TNBC patients in the training set.

Variables	Total (n = 352)	Non-RR-TNBC (n = 276)	RR-TNBC (n = 76)	*p*-Value
Age at diagnosis				0.054
<50 years	174 (49.43)	129 (46.74)	45 (59.21)	
≥50 years	178 (50.57)	147 (53.26)	31 (40.79)	
Menstrual status				0.284
Premenopausal	194 (55.11)	148 (53.62)	46 (60.53)	
Postmenopausal	158 (44.89)	128 (46.38)	30 (39.47)	
Family history				0.107
No	322 (91.48)	249 (90.22)	73 (96.05)	
Yes	30 (8.52)	27 (9.78)	3 (3.95)	
Surgical approach				0.091
Radical surgery	321 (91.19)	248 (89.86)	73 (96.05)	
Breast-conserving surgery	31 (8.81)	28 (10.14)	3 (3.95)	
Pathological pattern				0.490
Invasive ductal carcinoma	339 (96.31)	267 (96.74)	72 (94.74)	
Metaplastic	8 (2.27)	5 (1.81)	3 (3.95)	
Others	5 (1.42)	4 (1.45)	1 (1.32)	
Postoperative pathological TNM staging				<0.001
0 + I	135 (38.35)	128 (46.38)	7 (9.21)	
II	119 (33.81)	100 (36.23)	19 (25.00)	
III	98 (27.84)	48 (17.39)	50 (65.79)	
Postoperative pathological T staging				<0.001
T0/Tis + T1	215 (61.08)	197 (71.38)	18 (23.68)	
T2	82 (23.30)	52 (18.84)	30 (39.47)	
T3+T4	55 (15.62)	27 (9.78)	28 (36.84)	
Postoperative pathological N staging				<0.001
N0	185 (52.56)	174 (63.04)	11 (14.47)	
N1	86 (24.43)	67 (24.28)	19 (25.00)	
N2	36 (10.23)	20 (7.25)	16 (21.05)	
N3	45 (12.78)	15 (5.43)	30 (39.47)	
Tumor grade				0.598
G1+G2	139 (39.49)	107 (38.77)	32 (42.11)	
G3	213 (60.51)	169 (61.23)	44 (57.89)	
Lymph-vascular invasion				0.637
No	62 (17.61)	50 (18.12)	12 (15.79)	
Yes	290 (82.39)	226 (81.88)	64 (84.21)	
sTIL expression levels				<0.001
Low	83 (23.58)	34 (12.32)	49 (64.47)	
Intermediate	100 (28.41)	78 (28.26)	22 (28.95)	
High	169 (48.01)	164 (59.42)	5 (6.58)	
Her2 expression levels				0.194
IHC 0	62 (17.61)	48 (17.39)	14 (18.42)	
IHC 1+	197 (55.97)	149 (53.99)	48 (63.16)	
IHC 2+/Fish-	93 (26.42)	79 (28.62)	14 (18.42)	
Ki67				0.616
Ki67 ≤ 20	76 (21.59)	58 (21.01)	18 (23.68)	
Ki67>20	276 (78.41)	218 (78.99)	58 (76.32)	
P53				0.256
Negative	187 (53.12)	151 (54.71)	36 (47.37)	
Positive	165 (46.88)	125 (45.29)	40 (52.63)	
CK5/6				0.875
Negative	81 (23.01)	63 (22.83)	18 (23.68)	
Positive	271 (76.99)	213 (77.17)	58 (76.32)	
EGFR				0.373
Negative	83 (23.58)	68 (24.64)	15 (19.74)	
Positive	269 (76.42)	208 (75.36)	61 (80.26)	
Radiation therapy status				0.839
No	117 (33.24)	91 (32.97)	26 (34.21)	
Yes	235 (66.76)	185 (67.03)	50 (65.79)	
Chemotherapy drugs used				0.750
Anthracyclines	2 (0.57)	2 (0.72)	0 (0.00)	
Taxanes	16 (4.55)	13 (4.71)	3 (3.95)	
Anthracyclines + taxanes	244 (69.32)	194 (70.29)	50 (65.79)	
Combined with platinum	90 (25.57)	67 (24.28)	23 (30.26)	
NAC response				<0.001
NR	111 (31.53)	46 (16.67)	65 (85.53)	
ORR	241 (68.47)	230 (83.33)	11 (14.47)	

RR-TNBC: Rapid relapse triple-negative breast cancer.

Non-RR-TNBC: Non Rapid relapse triple-negative breast cancer.

### 3.3 Multivariate analysis of clinical and pathological parameters in neoadjuvant therapy triple-negative breast cancer patients with rapid relapse *versus* non-rapid relapse in the training set

To further elucidate the associations between clinical and pathological parameters in TNBC patients with rapid relapse *versus* non-rapid relapse, variables with a significance level of *P* < 0.10 from the univariate analysis were included in a multivariate logistic stepwise regression. The multivariate analysis revealed that age at diagnosis (age ≥50 years, OR = 0.325, 95% CI: 0.137–0.771), postoperative pathological N staging (N3 stage, OR = 13.669, 95% CI: 3.693–50.592),sTIL expression (sTIL moderate expression, OR = 0.272, 95% CI: 0.109–0.678; sTIL high expression, OR = 0.169, 95% CI: 0.048–0.594), and NAC response (ORR, OR = 0.059, 95% CI: 0.024–0.143) were predictive factors for rapid relapse in NAC TNBC patients (Table 3).

**TABLE 3:** Multivariate analysis of clinical and pathological parameters in neoadjuvant chemotherapy triple-negative breast cancer patients with rapid relapse *versus* non-rapid relapse in the training set.

Variables	Estimate	Se	z	Wald	*p*-Value	OR (95%CI)
(Intercept)	0.626	0.540	1.158	1.341	0.247	
Age at diagnosis						
<50 years	ref					
≥50 years	−1.124	0.441	−2.551	6.507	0.011	0.325 (0.137, 0.771)
sTIL expression levels						
Low	ref					
Intermediate	−1.302	0.466	−2.793	7.800	0.005	0.272 (0.109, 0.678)
High	−1.778	0.642	−2.771	7.679	0.006	0.169 (0.048, 0.594)
NAC response						
NR	ref					
ORR	−2.831	0.450	−6.288	39.544	<0.001	0.059 (0.024, 0.143)
Postoperative pathological N staging						
N0	ref					
N1	0.854	0.525	1.629	2.653	0.103	2.350 (0.840, 6.571)
N2	0.809	0.609	1.328	1.763	0.184	2.245 (0.680, 7.405)
N3	2.615	0.668	3.917	15.341	<0.001	13.669 (3.693, 50.592)

### 3.4 Establishment of nomogram prediction model

**FIGURE 2:**
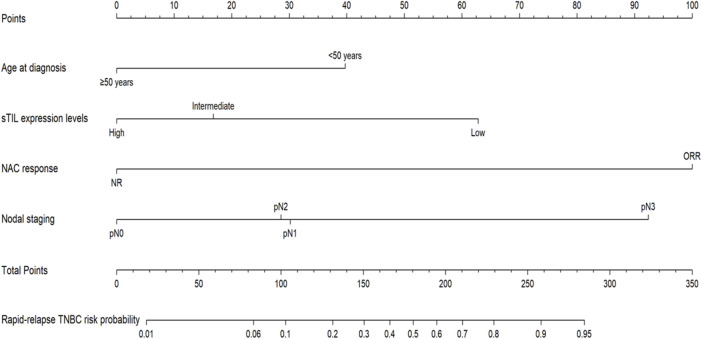
Nomogram prediction model for rapid relapse in NAC TNBC patients.

Utilizing R language on predictive factors selected through Logistic regression screening, a nomogram prediction model for rapid relapse in NAC TNBC patients was developed, with results shown in Figure 2. The first row in the chart corresponds to the scores of various descriptive parameters, while the second to fifth rows list the predictive factors: age at diagnosis, sTIL expression levels, NAC response, and N staging. Based on actual clinical and pathological indicators, scores for different parameters are obtained from the first row, and by summing the scores of all factors, the total score is calculated (sixth row). Finally, projecting this total score vertically yields the probability of rapid relapse in NAC TNBC patients (seventh row).

### 3.5 Evaluation of the prediction model

#### 3.5.1 ROC (receiver operating characteristic) curve

**FIGURE 3:**
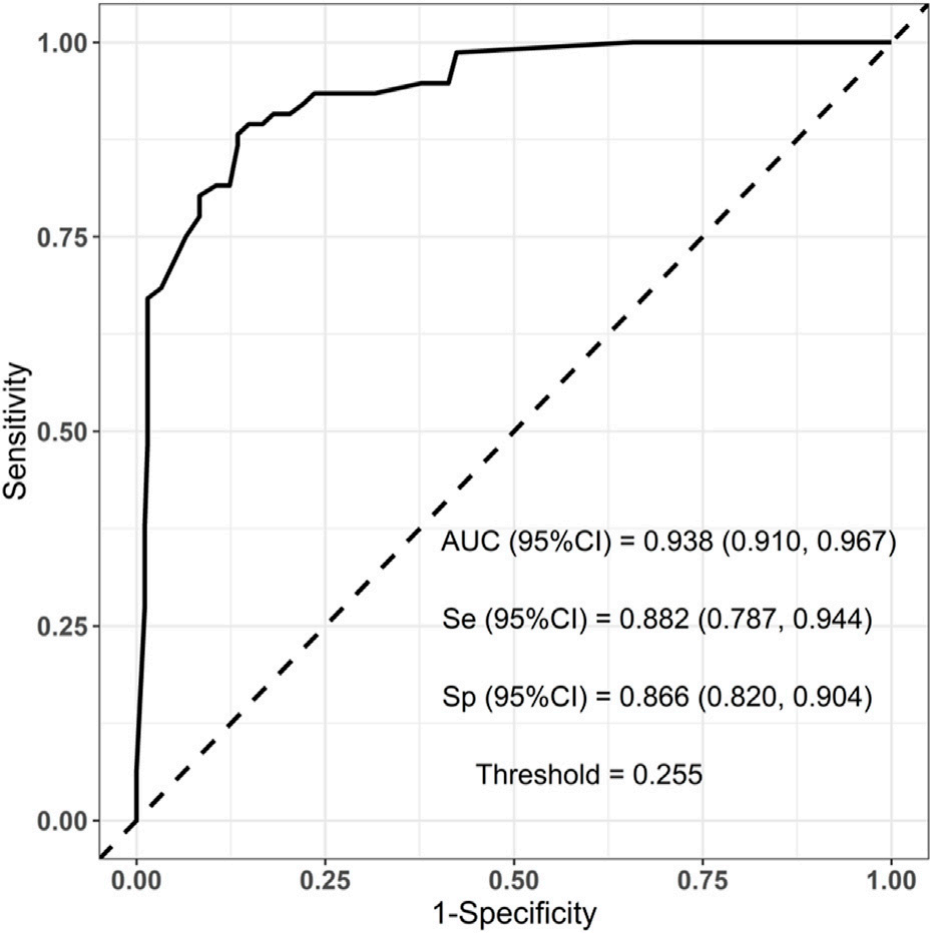
Training set ROC curve: Auc = 0.938 (95% CI = 0.910–0.967).

**FIGURE 4:**
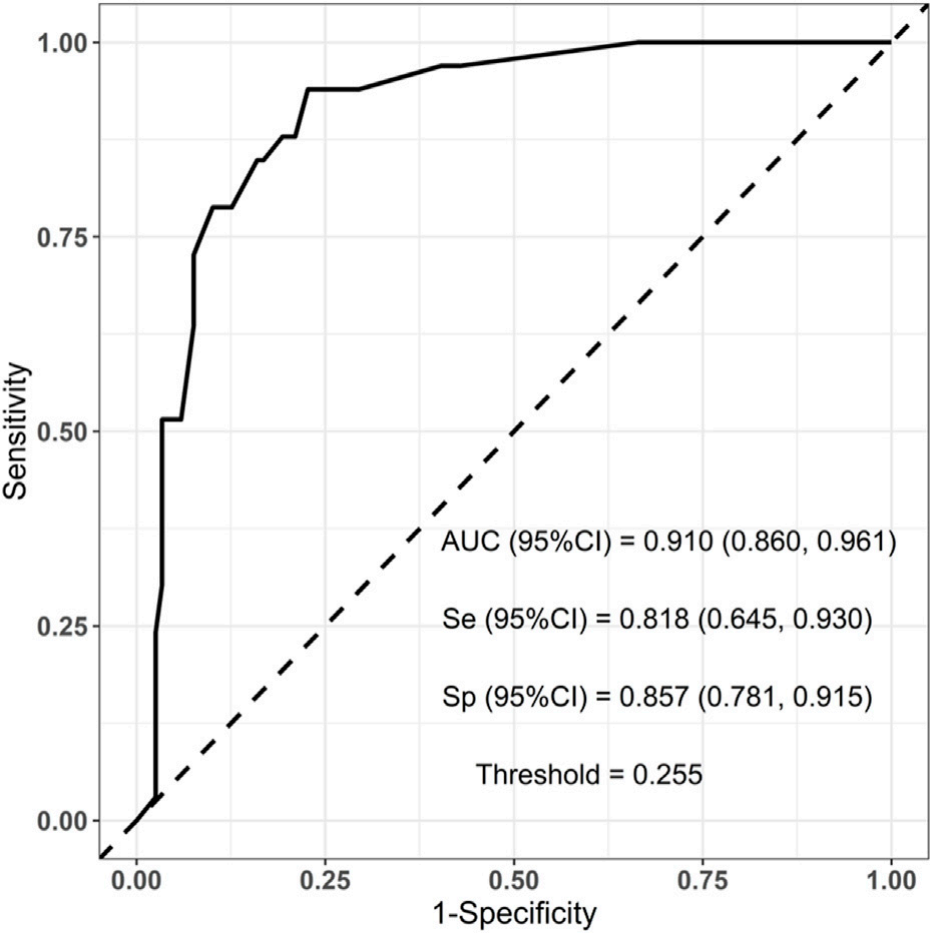
Validation set ROC curve: Auc = 0.910 (95% CI = 0.860–0.961).

To evaluate the established prediction model, ROC curves were plotted and AUC values were calculated for both the training and validation sets. The AUC values for the training and validation sets were 0.938 and 0.910, respectively, indicating a good predictive capability of the model as shown in Figures 3, 4.

#### 3.5.2 Calibration curve

**FIGURE 5:**
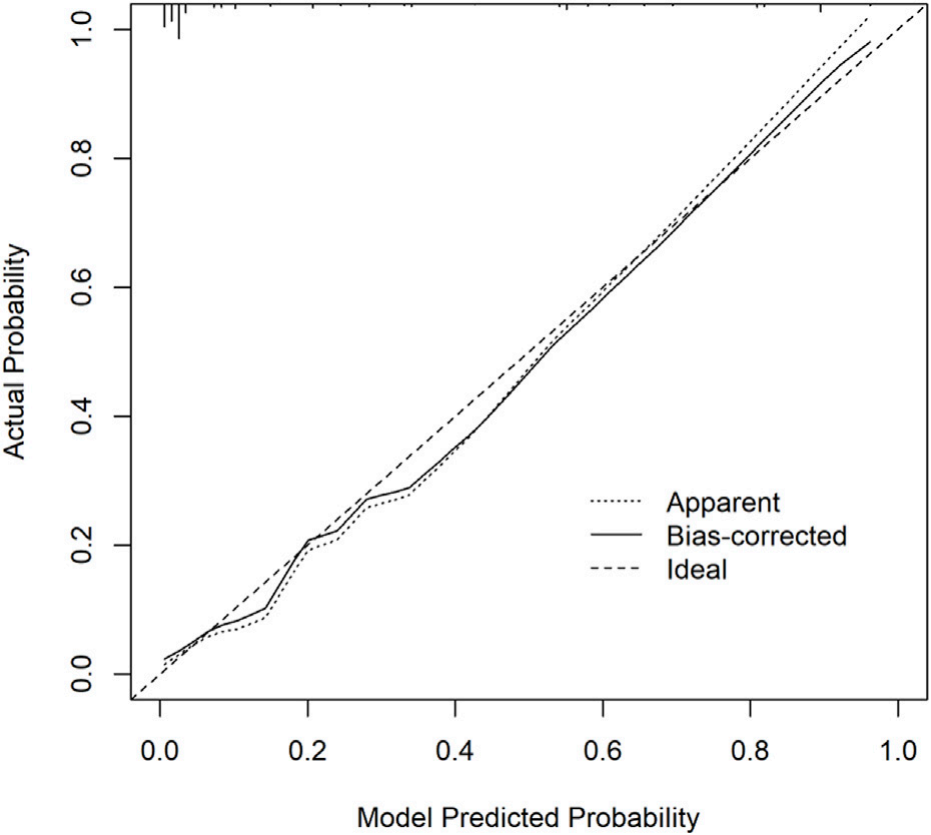
Training set calibration curve. The *x*-axis represents the model predicted probability, while the *y*-axis represents the actual probability.

**FIGURE 6:**
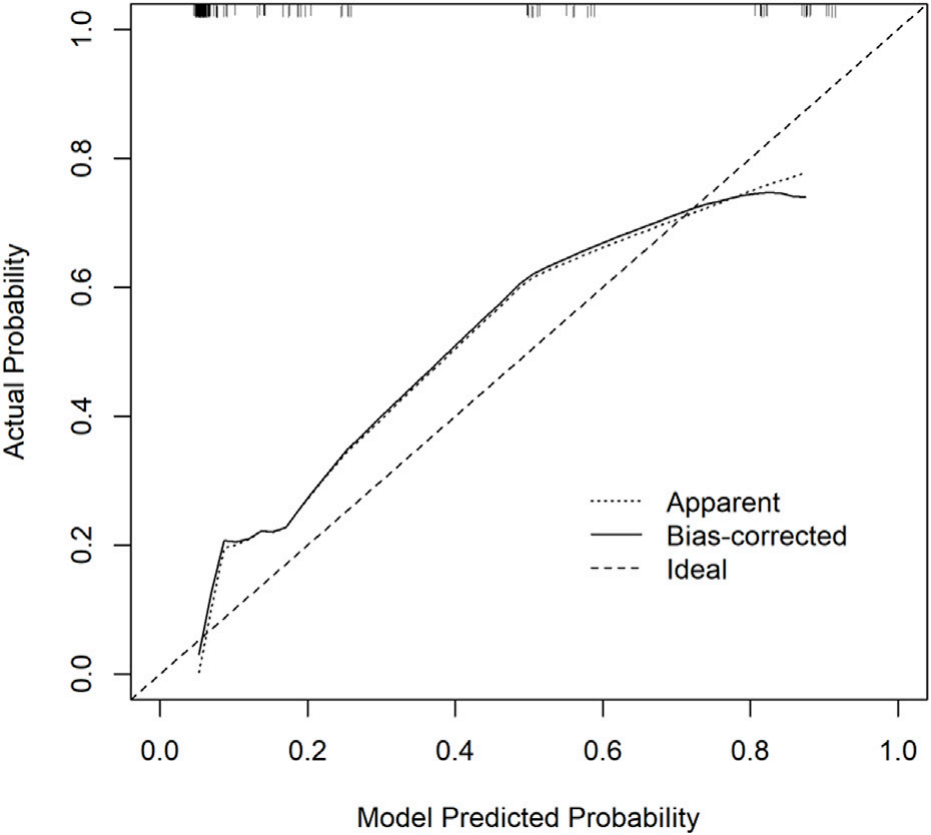
Validation Set Calibration Curve. The *x*-axis represents the model predicted probability, while the *y*-axis represents the actual probability.

Calibration curves were plotted on both the training and validation set samples to validate the model, with results depicted in Figures 5, 6.

#### 3.5.3 Decision curve analysis (DCA)

**FIGURE 7:**
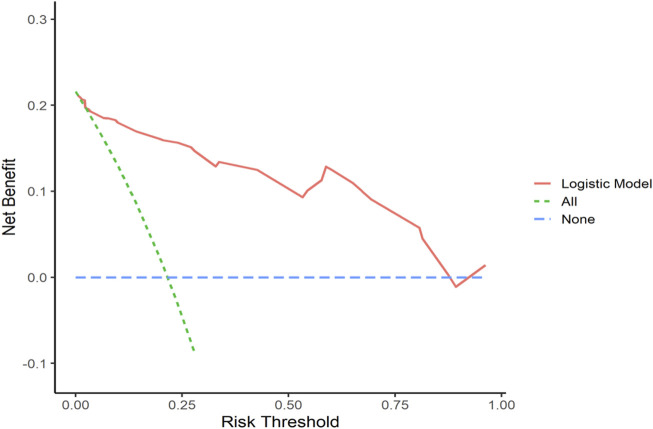
Training Set DCA Curve Net benefit is represented on the *y*-axis, while the threshold probability is shown on the *x*-axis. The red line represents the predictive model, the green line (“All”) represents the hypothesis of rapid relapse in NAC TNBC patients, and the blue line (“None”) represents the hypothesis of no rapid relapse in NAC TNBC patients.

**FIGURE 8:**
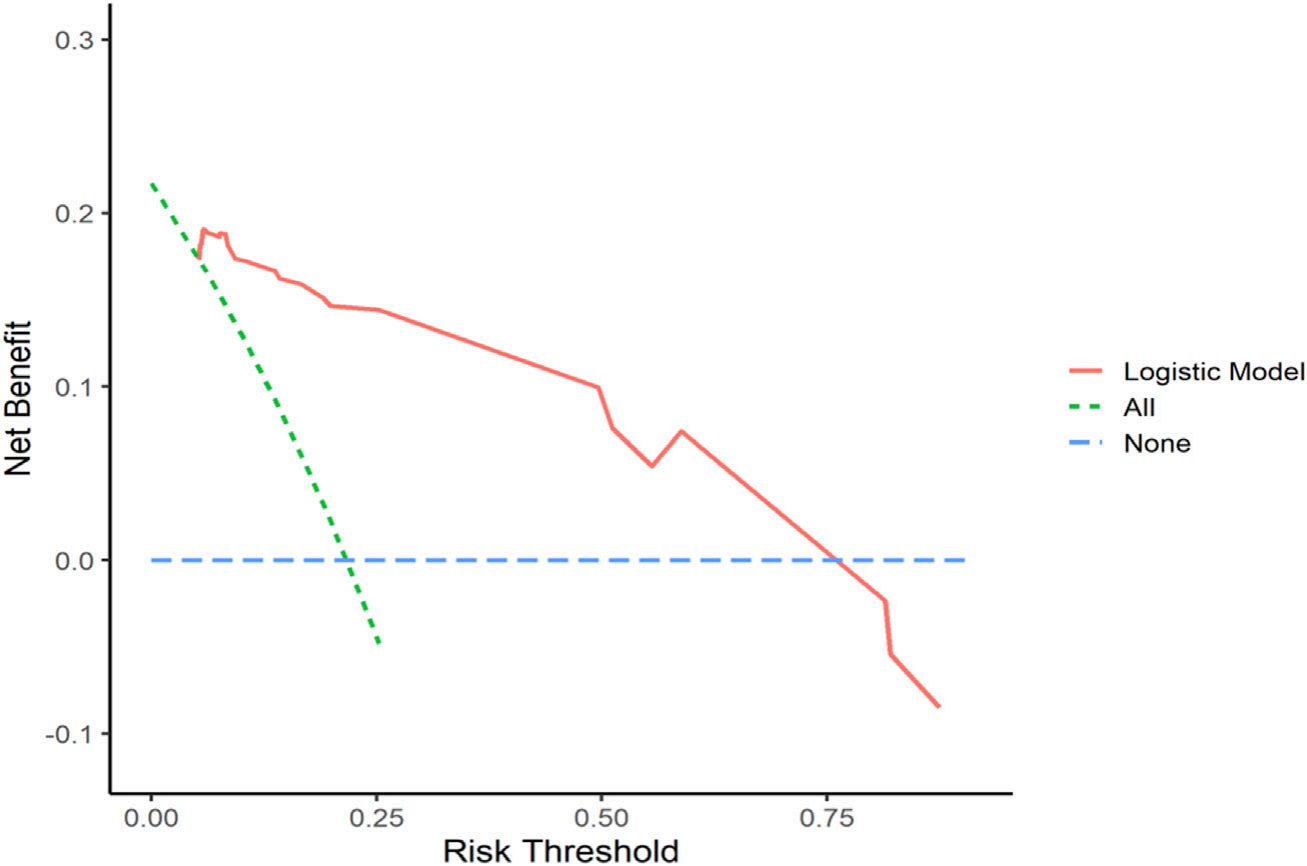
Validation Set DCA Curve Net benefit is represented on the *y*-axis, while the threshold probability is shown on the *x*-axis. The red line represents the predictive model, the green line (“All”) represents the hypothesis of rapid relapse in NAC TNBC patients, and the blue line (“None”) represents the hypothesis of no rapid relapse in NAC TNBC patients.

Decision curve analysis curves were plotted to validate the model, with results shown in Figures 7, 8.

## 4 Discussion

As is well known, TNBC is a highly heterogeneous disease. While there are many predictive models for recurrence and metastasis risk factors in TNBC, research on rapid relapse of TNBC in the neoadjuvant chemotherapy field is almost nonexistent. Therefore, the findings of this study will help clinicians further identify this subset of patients. By enhancing close follow-up or intensifying treatment, it is hoped that the survival outcomes of these patients could be improved.

Previous research suggests that patients with poorer pathological types, vascular invasion, higher tumor grades, increased Ki67 expression levels, advanced T staging, and N staging are more prone to postoperative recurrence and metastasis, leading to worse prognosis (Shi et al., 2019; Yang et al., 2019; Elimimian et al., 2021; Kim et al., 2020; Tadros et al., 2021). However, in our univariate and multivariate analysis, we found that only N3 staging is a risk factor for rapid relapse in neoadjuvant chemotherapy for TNBC. This finding aligns with prior related research (Laura et al., 2006; Xie et al., 2021; Geurts et al., 2017). Moreover, patients with N3 staging have a 13.669 times higher risk of rapid relapse compared to patients with N0 staging (N3 staging, OR = 13.669, 95% CI: 3.693–50.592), suggesting that as the number of lymph node metastases increases postoperatively, the risk of rapid recurrence and metastasis in neoadjuvant-treated TNBC patients also rises. However, we did not observe correlations between pathological patterns, tumor grade, presence of vascular invasion, Ki67 expression levels, T staging, and rapid relapse in neoadjuvant chemotherapy for TNBC in our multivariate analysis. One possible explanation is that lymphatic metastasis, being one of the most common routes of metastasis in breast cancer patients, signifies a more dangerous signal due to the potential for hematogenous spread and distant organ involvement following regional lymph node metastasis. Therefore, lymph node metastasis, especially when occurring in larger numbers, poses a higher risk of further distant metastasis, particularly in cases where systemic treatment efficacy is suboptimal.

Daniel G. Stover and colleagues’ report indicates a significant correlation between rapid relapse in triple-negative breast cancer (TNBC) and factors such as patient reliance on medical assistance/poverty insurance, low income, and diagnosis of breast cancer at a younger age (<50 years old) (Asad et al., 2021). However, within the realm of neoadjuvant chemotherapy, there is currently little literature reporting the relationship between rapid relapse in TNBC and sociodemographic factors. Therefore, in our study, we also considered some sociodemographic factors to analyze their relationship with rapid relapse in TNBC. Through univariate and multivariate analyses in our research, we observed that menstrual status and family history showed no significant correlation with rapid relapse in neoadjuvant-treated TNBC. However, being diagnosed with breast cancer at an age of ≥50 years emerged as a protective factor against rapid relapse in neoadjuvant-treated TNBC. The risk of rapid relapse in patients aged ≥50 years was 0.325 times that of patients aged <50 years (age≥50 years, OR = 0.325, 95% CI: 0.137–0.771), indicating that patients aged ≥50 years are less likely to experience postoperative rapid relapse, which is more common in patients under 50 years old. This finding aligns with previous related research and may be linked to the genomic instability and increased biological aggressiveness seen in younger TNBC patients.

The mainstream view in academia has long held that the efficacy of neoadjuvant chemotherapy is the best prognostic biomarker for TNBC patients (Schmid et al., 2022; Cortazar et al., 2014; Huang et al., 2020; Geyer et al., 2022). Therefore, this study incorporated the efficacy of neoadjuvant chemotherapy into the parameters analyzed for rapid relapse in TNBC. Through univariate and multivariate analyses in this study, it was found that the effectiveness of neoadjuvant chemotherapy serves as a protective factor against rapid relapse in TNBC. In other words, patients with poor neoadjuvant treatment efficacy are more prone to rapid relapse. Furthermore, the risk of rapid relapse in patients with effective neoadjuvant chemotherapy is 0.059 times that of patients with ineffective neoadjuvant chemotherapy (effective neoadjuvant therapy, OR = 0.059, 95% CI: 0.024–0.143). This study’s findings once again validate that the efficacy of neoadjuvant chemotherapy is a valuable predictive and prognostic indicator, particularly for highly aggressive subgroups such as triple-negative breast cancer. Regarding the potential significant correlation between different surgical approaches for breast cancer, various chemotherapy agents, radiotherapy, and neoadjuvant therapy in relation to rapid relapse in TNBC, our study did not find such associations through univariate and multivariate analyses in the neoadjuvant chemotherapy domain. This differs slightly from previous related research (Gradishar et al.,2024; Recht et al., 2016), indicating that further cross-center data and larger sample sizes are likely needed for internal and external validation.

Besides that,our study also included an analysis of the relationship between different Her2 expression levels and rapid relapse in neoadjuvant-treated TNBC. Through univariate and multivariate analyses in the neoadjuvant chemotherapy domain, our study did not find a correlation between different Her2 expression levels and rapid relapse in neoadjuvant-treated TNBC. This lack of association may be attributed to the fact that in the neoadjuvant therapy field since most patients undergo preoperative neoadjuvant chemotherapy, tumor staging tends to be somewhat advanced. As a result, compared to other clinical pathological indicators, the association between different Her2 expression levels and rapid relapse in neoadjuvant-treated TNBC may not be as pronounced. However, we believe that the connection between different Her2 expression levels and rapid relapse in neoadjuvant-treated TNBC is still inconclusive, and further research for validation analysis may be necessary.

In addition, we included relevant parameters related to the tumor microenvironment to investigate whether they affect the occurrence of rapid relapse in neoadjuvant-treated TNBC. Although some studies have found that TNBC patients expressing sTIL enrichment are more prone to postoperative recurrence and metastasis after receiving neoadjuvant therapy (Giacchetti et al., 2022), our study, through univariate and multivariate analysis, found that moderate and high expression of sTILs are protective factors against rapid relapse in neoadjuvant-treated TNBC. This finding aligns with the majority of research reports (Park et al., 2019; Gao et al., 2020; T de Jong et al., 2022; Leon-Ferre et al., 2024; De Boo et al., 2024). Moreover, the risk of rapid relapse for patients with moderate and high sTIL expression is 0.272 and 0.169 times that of patients with low sTIL expression, respectively (moderate sTIL expression, OR = 0.272, 95% CI: 0.109–0.678; high sTIL expression, OR = 0.169, 95% CI: 0.048–0.594). The primary reason for this may be that TNBC patients with enriched sTIL expression tend to have stronger anti-tumor immune capabilities. Furthermore, in our study, we did not find significant correlations between positive expressions of P53, CK5/6, and EGFR and rapid relapse in neoadjuvant-treated TNBC. This differs slightly from previous related research (Foulkes et al., 2010; Pan et al., 2017). We believe that further validation may require larger sample sizes and even data spanning different populations and centers.

In our study, several limitations should be considered: 1. The main limitations include heterogeneous TNBC patients and a retrospective study from a single center. 2. The absence of a thorough preoperative workup is a limitation in our study, particularly for TNBC patients with obvious lymph node involvement (N2 or N3 disease) or poor response to neoadjuvant therapy. We hope to identify these patients through larger sample sizes and more precise preoperative screening methods in the future (such as the breast MRI examination, chest and cranium CT examination, etc.), so that we can exclude those who have pre-existing disease. This will allow our algorithm to more accurately predict relapse in patients who are free of metastatic disease at presentation and ostensibly nodal early disease after neoadjuvant chemotherapy.3. The lack of more demographic, genomic data, and detailed treatment information. 4. In recent years, continuous updates in some pathological assessment methods may lead to diagnostic and selection errors. 5. The lack of external validation in other breast treatment center patients for the generalizability of the prediction model.

## 5 Conclusion

Finally, we constructed a nomogram model based on logistic regression to identify predictive factors within clinical-pathological features for rapid relapse in neoadjuvant-treated TNBC patients. This nomogram model was visualized using a column chart. Validation of the model was conducted through ROC curves, calibration curves, and decision curve analysis. The C-index values for the training and validation sets were 0.938 and 0.910, respectively. Additionally, the Brier scores for the training and validation sets were 0.076 and 0.097, indicating that the model demonstrates good discrimination and calibration in predicting rapid relapse in neoadjuvant-treated TNBC patients.

## Data Availability

The original contributions presented in the study are included in the article/supplementary material, further inquiries can be directed to the corresponding authors.
